# A curated diverse molecular database of blood-brain barrier permeability with chemical descriptors

**DOI:** 10.1038/s41597-021-01069-5

**Published:** 2021-10-29

**Authors:** Fanwang Meng, Yang Xi, Jinfeng Huang, Paul W. Ayers

**Affiliations:** Department of Chemistry and Chemical Biology, McMaster University, Hamilton, L8S 4L8 Canada

**Keywords:** Cheminformatics, Computational chemistry, Drug delivery, Virtual screening

## Abstract

The highly-selective blood-brain barrier (BBB) prevents neurotoxic substances in blood from crossing into the extracellular fluid of the central nervous system (CNS). As such, the BBB has a close relationship with CNS disease development and treatment, so predicting whether a substance crosses the BBB is a key task in lead discovery for CNS drugs. Machine learning (ML) is a promising strategy for predicting the BBB permeability, but existing studies have been limited by small datasets with limited chemical diversity. To mitigate this issue, we present a large benchmark dataset, B3DB, complied from 50 published resources and categorized based on experimental uncertainty. A subset of the molecules in B3DB has numerical log *BB* values (1058 compounds), while the whole dataset has categorical (BBB+ or BBB−) BBB permeability labels (7807). The dataset is freely available at https://github.com/theochem/B3DB and 10.6084/m9.figshare.15634230.v3 (version 3). We also provide some physicochemical properties of the molecules. By analyzing these properties, we can demonstrate some physiochemical similarities and differences between BBB+ and BBB− compounds.

## Abstract (summary)

**Table:** 

Measurement(s)	blood-brain barrier permeability
Technology Type(s)	digital curation
Sample Characteristic - Organism	organic molecules

Machine-accessible metadata file describing the reported data: 10.6084/m9.figshare.16632412

## Background & Summary

The blood-brain barrier (BBB) denotes a regulatory and protective mechanism of microvasculature in the central nervous system (CNS) that is central to regulating the homeostatis of the CNS^1,2^ and protecting the CNS from toxins, pathogens, and inflammations^3^. However, it is estimated that 98% of small molecule drugs are not BBB permeable^4^. Therefore, predicting BBB permeability for small molecules is a vital but challenging task in drug discovery and development^4–7^. However, existing computational models for a molecule’s BBB permeability are inadequate. In particular, they are restricted by the limited size and chemical diversity of existing sets of training data^8^. Moreover, although many different machine-learning (ML) models for predicting BBB permeability have been proposed, these models are not directly comparable because they use widely varying training data, ranging from as few as 45 molecules^9,10^ to as many as 7236 molecules^11^. The purpose of this paper is to curate an accessible, clean, well-documented, and reasonably comprehensive dataset of BBB permeability data and present it in a way that is convenient for those building new BBB predictive models. While our database, B3DB, is not the first attempt to curate data from the literature to construct a molecular BBB database, B3DB contains more molecules, and categorizes the molecules based on experimental uncertainty. Both features are very helpful when developing and validating ML models for BBB.

There are two types of data for BBB, numerical and categorical data. Numerical data is usually reported as log *BB*, the logarithm of brain-plasma concentration ratio,

1 $$\log \;BB=\log \frac{{C}_{brain}}{{C}_{blood}}$$

Categorical data simply labels whether a compound is BBB permeable (BBB+) or not (BBB−).

Among existing studies of BBB permeability, we mention Zhuang *et. al*., who built a ML model with resampling using a binary dataset of 2358 molecules^12^. Similarly, Zhao *et al*.^13^ compiled a dataset of 1336 BBB crossing drugs (BBB+) and 360 BBB non-crossing drugs (BBB−). To our knowledge, the largest dataset previously reported in the literature was used in developing the lightBBB model, which uses the Light Gradient Boosting Machine (LightGBM) algorithm to build a predictive model. The lightBBB model’s database included 7162 entries. (These entries include duplicates (multiple entries with the same International Chemical Identifier (InChI)) and molecules that could not be recognized by RDKit, so in the end there are only 4491 unique valid molecules). We curate data from these three efforts, and 47 other smaller efforts, in B3DB. Unlike many previous efforts, B3DB includes many (1058) molecules with numeric log *BB* values. The largest previous dataset we know was the data source for lightBBB, which has log *BB* values for 696 unique valid molecules.

Here, we present a new Blood-Brain Barrier Database, B3DB, which is intended to provide a benchmark dataset for modelling BBB permeability of small molecules. The original data was collected from 50 peer-reviewed publications or open access datasets. As described in the next section, we processed and cleaned the data, then categorized it based on its reliability. By categorizing the data in this way, users can choose whether they want to focus on the smaller subsets with the highest reliability, or prefer to consider larger datasets with slightly lower reliability. We hope that our meticulous methods of preparing and sorting the data may be of interest those who wish to curate databases for other, similar, properties.

B3DB includes both numerical data (1058 log *BB* values) and categorical data (4956 BBB+ and 2851 BBB−). Here is summary of key features of B3DB dataset. (1) This is the largest BBB data set we know, both for categorical labels and log *BB* numerical values. (2) Because the chirality of molecules plays an important role in BBB permeability^14,15^, isomeric SMILES is to used to incorporate chiral specifications of molecules. (3) Because some molecules have been measured multiple times, using different experimental methods and under different conditions, we divide the value into groups based on the quantity of experimental data and the similarity between reported values, so that users of B3DB can easily select subsets of the data with varying degrees of reliability. (4) B3DB is extended with molecular descriptors computed with mordred^16^, so that it can be used out-of-the-box for building BBB predictive models.

## Methods

**Fig. 1:**
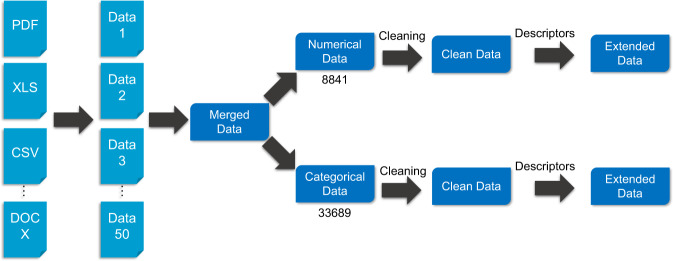
Workflow for building B3DB. From left to right, the collection of raw BBB data, cleaning the raw data, categorization of cleaned data, and finally, extension of B3DB by computing other molecular descriptors.

The next three sections describe how raw data was collected from various sources, cleaned, and curated. We then describe how the dataset was extended with chemical descriptors (beyond the reference BBB value). This workflow is summarized in Fig. 1. For consistency and reproducing purposes, all the data processing were performed in a Python 3.7.9 virtual environment created with Conda in CentOS Linux release 7.9.2009 which include pandas 1.2.1, tabula-py 2.2.0, RDKit 2020.09.1, pubchempy 1.0.4, OEChem Toolkit^17^ provided by openeye-toolkit 2020.2.0, ChEMBL_Structure_Pipeline 1.0.0, SciPy 1.5.2, Numpy 1.19.2, mordred 1.1.1, PyTDC 0.1.5. ALOGPS version 2.1 is also used for calculating octanol/water partition coefficient log *P*.

### Data collecting

All the data was collected from the literature and open source databases. The dataset size, main available information, and data types are listed in Table 1. For each data source, a standard Excel workbook is formatted for further processing. If the original data is in portable document format (PDF), it is converted to a pandas^18^. DataFrame and then stored in XLSX format with tabula-py^19^. For files in DOCX or DOC extension, as well as CSV, TXT and other Excel compatible formats, they are converted to Excel XLSX format directly, using Microsoft Office. We performed several automated consistency checks (e.g., numerical data should be reported as floating-point numbers) and manually verified a subset of the data to ensure that the data was faithfully transferred to *.xlsx format. In total, 33825 raw data records were collected.

**Table 1:** Data source and the available corresponding information.

ID	Data Source Size	Information Available	Data Type	Reference
R1	2053	name, smiles	categorical data	^28^
R2	1210	name, smiles	categorical data, numerical data	^32^
R3	328	name, smiles	numerical data	^35^
R4	189	CAS, name, smiles	numerical data	^36^
R5	108	name, smiles	numerical data	^37^
R6	1692	name, smiles	categorical data	^38^
R7	224	name	categorical data	^29^
R8	439	smiles, CID	numerical data	^25^
R9	415	name, smiles	categorical data	^39^
R10	462	name, CID	categorical data	^40^
R11	151	name, logBB	numerical data	^41^
R12	182	name, smiles	numerical data	^42^
R13	2321	smiles	categorical data	^12^
R14	942	name, smiles	categorical data	^43^
R15	390	name	categorical data	^44^
R16	374	name, CID	categorical data	^30^
R17	55	name	numerical data	^45^
R18	332	name, smiles	numerical data	^27^
R19	1990	name, smiles	categorical data	^13^
R20	139	name	numerical data	^46^
R21	362	name, smiles, CID	numerical data	^47^
R22	27	name	numerical data	^48^
R23	1090	name, smiles	categorical data	^49^
R24	1866	smiles	categorical data	^50^
R25	581	name, smiles	numerical data	^26^
R26	448	CAS, name, smiles	categorical data, numerical data	^51^
R27	7236	smiles	categorical data, numerical data	^11^
R28	415	name, smiles	categorical data	^31^
R29	181	name	categorical data	^52^
R30	3620	name, smiles	categorical data	^53^ *
R31	12	name	numerical data	^54^
R32	26	name	numerical data	^55^
R33	26	name	numerical data	^56^
R34	153	name	numerical data	^57^
R35	145	smiles	numerical data	^58^
R36	525	name, smiles	categorical data	^59^
R37	111	name, smiles	categorical data	^60^
R38	291	name, smiles	numerical data	^61^
R39	122	name	numerical data	^62^
R40	405	name	numerical data	^63^
R41	296	smiles	numerical data	^64^
R42	45	smiles	numerical data	^9^
R43	328	name, smiles	numerical data	^65^
R44	89	name	numerical data	^66^
R45	8	smiles	numerical data	^67^
R46	483	smiles	numerical data	^68^
R47	529	name	numerical data	^69^
R48	115	smiles	numerical data	^70^
R49	181	name, smiles	numerical data	^71^
R50	113	name, smiles	categorical data, numerical data	^72^

*Data accessed with PyTDC 0.1.5 as of Jan 25, 2021.

The 50 datasets have various formats and include a wide range of information, so we constructed a template that contained only the most essential data, compound name, simplified molecular-input line-entry system (SMILES) string, PubChem compound identifier (CID), log *BB*, BBB+/BBB− (whether a compound is BBB permeable or not), the IUPAC International Chemical Identifier (InChI), the threshold value used to determine categorical type of a compound, and the literature source for that data value.

### Data cleaning

**Fig. 2:**
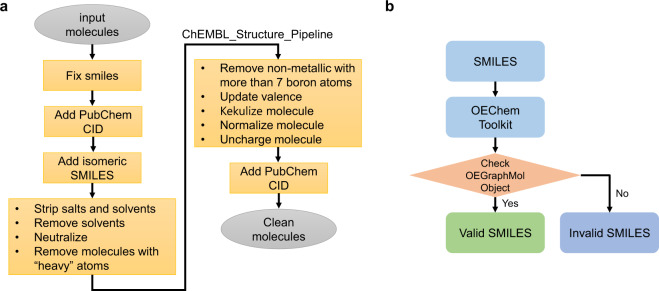
Molecule representation cleaning and technical validation. (**a**) Flowchart of cleaning SMILES string representation of molecules. (**b**) Technical validation of molecular representation.

In the data cleaning stage, an initial molecule specification (a SMILES string, PubChem CID, and/or compound name) is input; the output is also a SMILES string, but with transcription and typographical errors fixed, and with salts/solvents removed. In addition, molecules containing heavy metal atoms are removed from the database. A followed up standardization of molecular reorientation is performed which include updating valences, kekulizing and normalizing molecules, and neutralizing molecular charges. The basic procedure is shown in Fig. 2(a).

The first step is to fix invalid SMILES strings. For example, white spaces and line breaks in SMILES were removed. Some other issues (e.g., where a dash was used in lieu of a negative sign for the molecular charge) were manually remedied. Our data is drawn from 50 distinct sources, and a full molecule specification is not always provided. For example, some sources list only the compound names (and not the SMILES strings or PubChem CIDs); other sources list only PubChem CIDs. In these cases, PubChemPy^20^ was used to access the PubChem^21^ database to retrieve information about missing compound names, SMILES strings and PubChem CIDs. When only the compound name was available, there can be multiple PubChem instances. If this were to happen, the first Pubchem instance is selected and a note is added to the database flagging the potential ambiguity. Fortunately this does not seem to occur in this specific database. There are also a few molecules for which only molecular structures, and not SMILES or compound names, are provided. In these cases we built the molecules manually and searched for the Pubchem CID and SMILES string with the PubChem web interface. All the SMILES strings were loaded into RdKit^22^ (version 2019.03.4) to build molecule objects. If the object is None, the SMILES is considered to be invalid. This leads to 33771 measured BBB instances.

Stereochemistry can play a significant role in a molecule’s BBB permeability because of transporters’ specific stereoselectivity^14,15^. However, there is no stereochemical information in SMILES strings. To add stereochemical information to SMILES, and to deal with generic SMILES strings that were technically valid but not in canonical form, the original SMILES were upgraded to isomeric SMILES by using PUG-REST API^23^ wherever possible. Otherwise, the canonical SMILES were retrieved from PubChem database with PUG-REST API^23^. The inclusion of stereochemical data about the molecules is an important, and (we believe) unique feature of B3DB.

Once the SMILES representations are fixed, ChEMBL_Structure_Pipeline^24^ was used to strip the salts and neutralize the charge. Molecules containing metal atoms or heavy atom with atomic number greater than 20 (e.g., Zinc, Bromine, Krypton, Iodine, and Xenon) were removed. Molecules with more than 7 boron atoms are also excluded due to problems of depicting borane compounds. Implicit valence and ring information were recomputed followed by kekulizing, normalization of molecules and molecular charges were neutralized. These revisions change the molecular structure, so the Pubchem CIDs were updated from the revised SMILES strings.

### Data curation

**Fig. 3:**
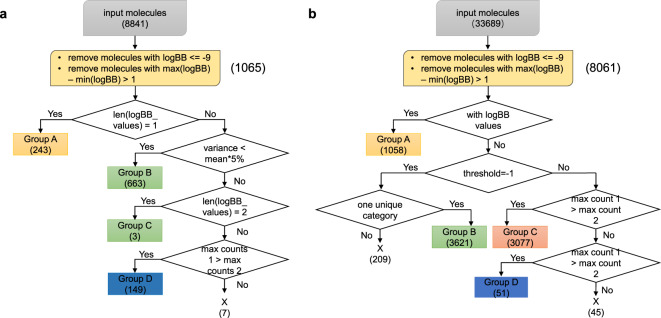
Curation algorithm for numeric and categorical BBB data. (**a**) Curation pipeline for BBB data with log *BB* values. (**b**) Curation pipeline for BBB data with categorical information, either BBB+ or BBB−.

The curation procedures for numerical and categorical data are summarized in Fig. 3. To curate the data, a unique chemical identifier is required. Although InChI is unique in principle, it cannot resolve tautomeric forms, which is a common source of ambiguity and error in chemical structure representation. Therefore, we examined the unique InChI generated with RdKit and the isomeric SMILES (and canonical SMILES where isomeric SMILES is unavailable). The number of unique SMILES is greater than the number of unique InChI values, but the redundancy is merely because each SMILES represents a specific resonance structure.

#### Curation of numerical data

To curate the 8841 numerical BBB data values, log *BB* values for each molecule were merged into a list. The 20 instances with log *BB* <= −9 were regarded as outliers because, based on the distribution of log *BB* values, they seemed suspicious. Next, we identified molecules where there are multiple reported log *BB* values and eliminated those molecules from the database if the reported values differed significantly. Specifically, we eliminated 16 molecules where max (log *BB*) − min(log *BB*) > 1. The values that remain after curation are merged into 1065 molecular records. The molecular records are augmented, as necessary, to ensure that they are complete, including compound name, IUPAC name, isomeric (canonical) SMILES, etc..

Here is the detailed curation procedure for numeric data.

1. **Group A** (243 molecules). Molecules with only one unique log *BB* value.
2. **Group B** (663 molecules). Molecules with more than one log *BB* value, but all the the reported values differ by less than 5% from the mean value. In these cases, the mean value is used as the log *BB* value for the molecule.
3. **Group C** (3 molecules). Other molecules with two distinct log *BB* values. The (weighted) mean value is used as the curated value for group C (just as for group B).
4. **Group D** (149 molecules). Other molecules with more than two distinct values; whichever value occurs with greatest frequency is used. In three case, two distinct values were reported with maximum frequency; we discarded those molecules from the dataset.

The 7 molecules which failed to be categorized as group A, B, C or D, they are discarded. The final dataset therefore contains 1058 molecules; for most of these molecules (815 molecules) multiple, mutually consistent, values of log *BB* are reported in the literature.

#### Curation of categorical data

The 33689 data values were divided into two categories, numerical data and (binary) categorical data.

1. **Group A** (1058 molecules). Molecules with numerical data. Several threshold values for log *BB* have been used to determine if a molecule is BBB permeable or not, including 0^25,26^, 0.1^27^, −1^12,13,28–31^, (−2, 1)^32^. The value of −1 is chosen as the threshold value to define if a compound is BBB+ or BBB− since this is the mostly widely used threshold and maximizes the ease of comparison with other studies.
2. **Group B** (3621 molecules). Molecules from sources that use log *BB* = −1 as the threshold value, and where all sources agree on the categorical label. The unambiguous label is used.
3. **Group C** (3077 molecules). Molecules where all sources agree on the categorical label, but the sources that do not report their threshold value.
4. **Group D** (51 molecules). Molecules with two different BBB permeability labels. The most prevalent label is used. In the 45 cases where the two labels occurred with equal frequency, the molecule was discarded.

The 7807 remaining molecular records are augmented to ensure that they are complete, including compound name, IUPAC name, isomeric (canonical) SMILES, etc..

### Data extension with chemical descriptors

To better facilitate building BBB predictive models, the curated datasets were extended with chemical descriptors. Then 1613 chemical descriptors were calculated with mordred version 1.1.1^16^. The purpose of providing this extended data is to facilitate easy use of the B3DB, without requiring precomputation of cheminformatics descriptors.

## Data Records

There are two datasets provided in this study, one with numeric log *BB* values (1058 molecules) and the other with categorical labels (7807 molecules with 4956 BBB+ and 2851 BBB−). B3DB data is stored in the comma-separated values (CSV) format and contains SMILES representations, compound name, IUPAC name, log *BB* value, threshold, BBB+/BBB− and the corresponding references along with 1613 molecular descriptors. This is summarized in Table 2. The data are openly accessible at GitHub (https://github.com/theochem/B3DB) as well as figshare platform^33^.

**Table 2:** List of information in the curated datasets.

Column Header	Description	Data Type
compound name	Generic name of compound	string
IUPAC name	Name of compound following the IUPAC nomenclature naming scheme	string
SMILES	SMILES representation of compound, isomeric SMILES if available	string
CID	PubChem compound identifier	string
log BB	log *BB* value of compound	float
BBB+/BBB−	Categorical labels to indicate if compound is BBB permeable (BBB+) or not (BBB−)	string
InChI	The IUPAC International Chemical Identifier of compound	string
threshold	Threshold value used to determine BBB permeability label	float
reference	Data sources	string
group	Group classification	string
comment	Complementary information	string

The BBB+/BBB− and threshold columns are only available for categorical dataset. The 1613 2D chemical descriptors are not listed in this table.

## Technical Validation

### Validation of molecular representations

All the molecules are in canonical SMILES format and, if available from PubChem, also isomeric SMILES. We then attempt to load each SMILES string into OEChem Toolkit^17^ as an OEGraphMol object; if this is successful then this SMILES is regarded as valid. (See Fig. 2(b)).

### Analysis of curated datasets

**Fig. 4:**
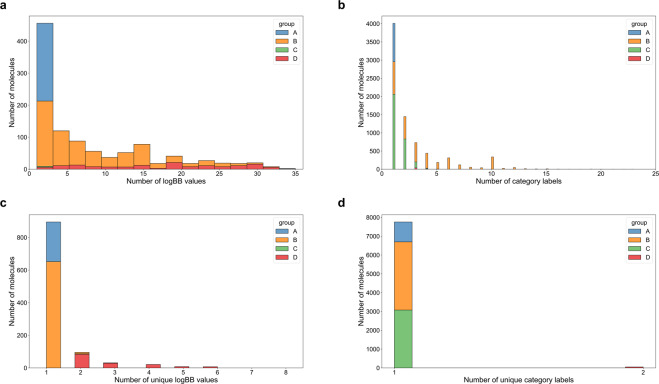
Characterization of the nature and frequency of multiple/redundant data in B3DB. (**a**) Multiplicity of source log *BB* values in each group of the numerical dataset. (**b**) Prevalence of source BBB permeability labels in each group of the categorical dataset. (**c**) Multiplicity of unique log *BB* values in each group of the numerical dataset. (**d**) Prevalence of unique BBB permeability labels in each group of the categorical dataset. More data can be found at Tables 3–6.

The BBB data comes from 50 sources, and was acquired in different laboratories, under different conditions, and using different protocols. To characterize the experimental uncertainty, we examine the agreement between reported values, Fig. 4. For 92.82% of the numerical data, there at most two unique log *BB* values are reported as shown in Fig. 4(a,c). Similarly, for 99.34% of the molecules, only a single categorical label is reported (Fig. 4(d)); this is true even though the same molecule may appear in as many as 23 distinct sources (Fig. 4(b)). More detailed data can be found in Tables 3–6.

**Table 3:** Occurrences of source log *BB* values for different groups in numerical dataset.

Group	A	B	C	D
Frequency				
1	243	0	0	0
2	0	32	3	0
3	0	172	0	6
4	0	38	0	7
5	0	71	0	4
6	0	46	0	8
7	0	29	0	5
8	0	19	0	3
9	0	29	0	5
10	0	13	0	4
11	0	17	0	3
12	0	19	0	3
13	0	26	0	4
14	0	58	0	6
15	0	8	0	6
16	0	5	0	0
17	0	10	0	3
18	0	6	0	11
19	0	4	0	7
20	0	10	0	3
21	0	4	0	5
22	0	6	0	3
23	0	7	0	4
24	0	9	0	7
25	0	4	0	5
26	0	7	0	3
27	0	2	0	6
28	0	5	0	5
29	0	3	0	10
30	0	1	0	6
31	0	1	0	3
32	0	1	0	3
33	0	1	0	0
34	0	0	0	0
35	0	0	0	1

**Table 4:** Occurrences of unique source log *BB* values for different groups in numerical dataset.

Group	A	B	C	D
Frequency				
1	243	652	0	0
2	0	9	3	84
3	0	2	0	29
4	0	0	0	21
5	0	0	0	8
6	0	0	0	7

**Table 5:** Occurrences of source BBB permeability labels for different groups in categorical dataset.

Group	A	B	C	D
Frequency				
1	1058	892	2062	0
2	0	618	831	0
3	0	533	162	37
4	0	409	17	13
5	0	181	5	1
6	0	313	0	0
7	0	121	0	0
8	0	55	0	0
9	0	41	0	0
10	0	338	0	0
11	0	26	0	0
12	0	47	0	0
13	0	15	0	0
14	0	8	0	0
15	0	11	0	0
16	0	3	0	0
17	0	2	0	0
18	0	1	0	0
19	0	3	0	0
20	0	1	0	0
21	0	1	0	0
22	0	1	0	0
23	0	1	0	0

**Table 6:** Occurrences of unique source BBB permeability labels for different groups in categorical dataset.

Group	A	B	C	D
Frequency				
1	1058	3621	3077	0
2	0	0	0	51

**Fig. 5:**
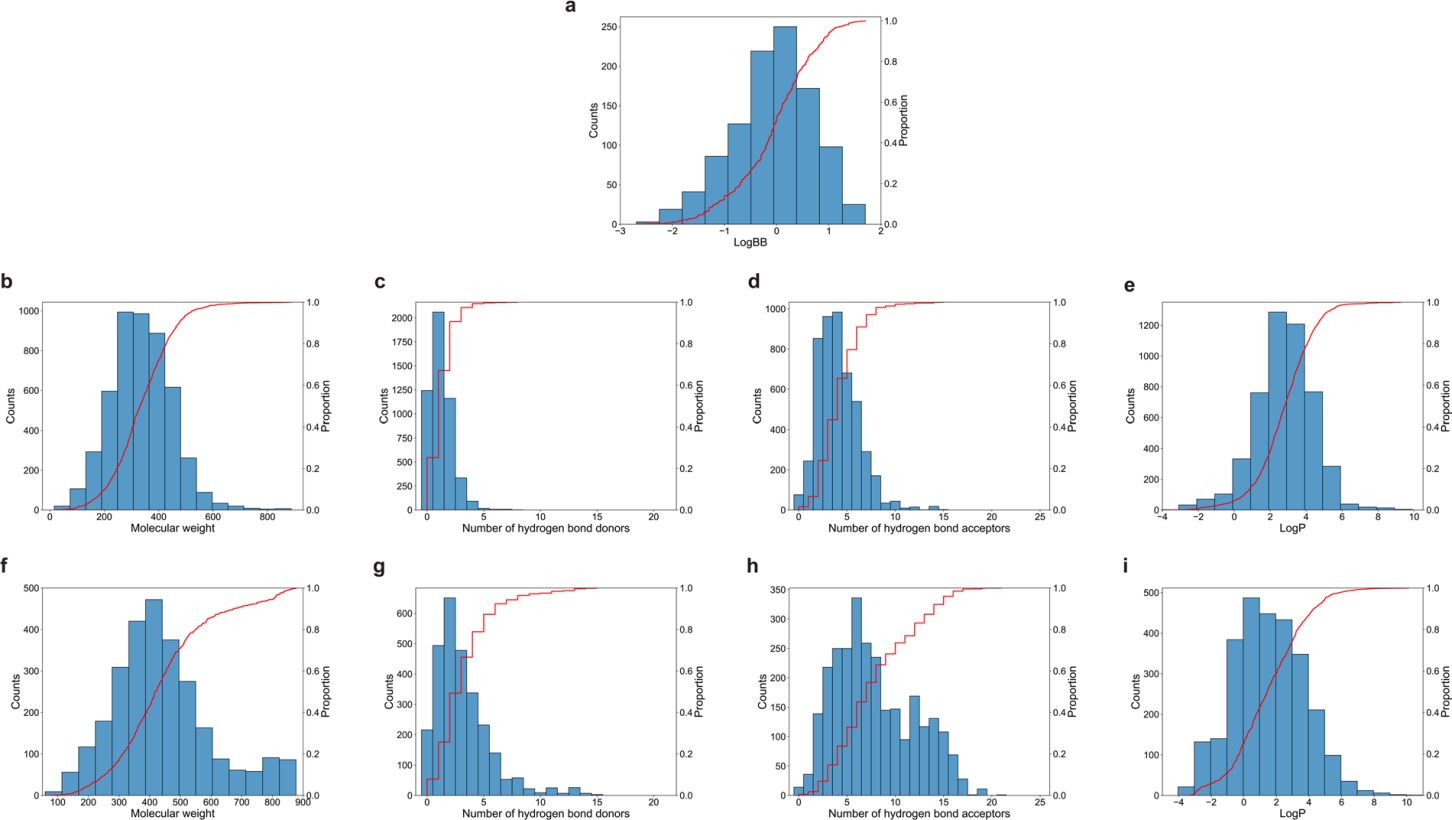
Analysis of the curated datasets. (**a**) Distribution of log *BB* values for numeric dataset. (**b**–**e**) Distribution of molecular weight, number of hydrogen-bond donors, number of hydrogen acceptors and log *P* for BBB+ compounds. (**f**–**i**) Distribution of molecular weight, number of hydrogen-bond donors, number of hydrogen acceptors and for BBB− compounds.

Figure 5 reveals some features of the B3DB dataset. Presuming that the molecules in the dataset are relatively representative of (bio)organic molecules in general, the log *BB* for most of organic compound lie within the interval [−2, 2] (see Fig. 5(a)). The distribution of log *BB* values indicates that the numerical dataset is relatively balanced, though skewed towards BBB+ compounds.

Lipinski’s Rule of 5 https://www.sciencedirect.com/science/article/abs/pii/S0169409X00001290?via%3Dihub is a simple rule-of-thumb for evaluating a molecule’s drug-likeness. Specifically, Lipinski’s Rule of 5 states that good absorption or permeation is more likely if a molecule has less than: 5 hydrogen-bond donors, 10 hydrogen-bond acceptors, 500 Dalton molecular weight, and a predicted log *P* value less than 5. It is observed that the molecule weight of most BBB+ compounds (93.10%) is less than 500 Dalton. In contrast, there are many molecules with molecular weight greater than 500 Dalton (31.22%) that are BBB− compounds. Nonetheless, aside from the a long tail of heavy BBB− compounds, the distribution of molecular weights for BBB+ and BBB− molecules is not dissimilar (see Fig. 5(b,f)). 98.8% of BBB+ compounds and 23.4% of BBB− compounds have fewer than 5 hydrogen-bond donors; 97.6% of BBB+ compounds and 66.0% of BBB− compounds have fewer than 10 hydrogen-bond acceptors. This supports the idea that hydrophilic compounds find it difficult to cross the BBB, but this is not a hard-and-fast rule: there are BBB+ compounds that violate Lipinski’s rule of 5. Finally, the octanol/water partition coefficient log *P* was estimated using ALOGPS version 2.1^34^. There is not much difference in the log *P* values for BBB+ and BBB− compounds: 93.8% of BBB+ and 95.1% of BBB− compounds have log *P* < 5. Taken together, the analysis of the selected physiochemical descriptors suggest that no single parameter can determine the BBB-permeability of a compound. This confirms that predicting BBB permeability computationally is challenging, and emphasizes the value of the B3DB dataset.

## Usage Notes

None of the original data sources contain any quantification of uncertainty (e.g., the standard derivation), so it is recommended to incorporate the group categories when using the datasets. If one decides to use a different threshold to determine BBB+ and BBB− for a molecules, log *BB* can be used directly from the data reported in this study. The 1613 2D chemical descriptors, computed with mordred can facilitate building predictive models. Any further molecular preprocessing can be done with RdKit.

## Data Availability

The codes used in this study have been deposited to https://github.com/theochem/B3DB and 10.6084/m9.figshare.15634230.v3 (version 3)^33^. All the calculation were done with Python 3.7.9 under a virtual environment created with Anaconda on Linux.
